# Kaempferol Alleviates Carbon Tetrachloride-Induced Liver Fibrosis in Mice by Regulating Intestinal Short-Chain Fatty Acids

**DOI:** 10.3390/ijms26146666

**Published:** 2025-07-11

**Authors:** Siqi Zhang, Fei Tang, Zhe Zhou, Linhui Li, Yang Tang, Kaiwen Fu, Yang Tan, Ling Li

**Affiliations:** 1 Hunan Key Laboratory of Integrated Chinese and Western Medicine for Prevention and Treatment of Heart and Brain Diseases, Hunan University of Chinese Medicine, Changsha 410208, China; zhangsiqi@stu.hnucm.edu.cn; 2 College of Pharmacy, Hunan University of Chinese Medicine, Changsha 410208, China; tf20010405@126.com (F.T.); shmily202506@163.com (Z.Z.); 20233759@stu.hnucm.edu.cn (L.L.); tangyang5255@163.com (Y.T.); fkwkko@163.com (K.F.); 004746@hnucm.edu.cn (Y.T.); 3 Key Laboratory of Modern Research of TCM, Education Department of Hunan Province, Hunan University of Chinese Medicine, Changsha 410208, China; 4 National Key Laboratory Cultivation Base of Chinese Medicinal Powder & Innovative Medicinal Jointly Established by Province and Ministry, Hunan University of Chinese Medicine, Changsha 410208, China

**Keywords:** liver fibrosis, kaempferol, CCl_4_, mice, gut microbiota, short-chain fatty acids, oxidative damage

## Abstract

Liver fibrosis remains a critical health concern with limited therapeutic options. Kaempferol (Kae) is a natural flavonoid widely present in natural plants, yet its role in modulating gut–liver axis interactions during fibrosis is unexplored. This study investigates the hepatoprotective effects of Kae on alleviating carbon tetrachloride (CCl_4_)-induced liver fibrosis, and its underlying mechanisms, focusing on oxidative stress, gut microbiota, and short-chain fatty acids (SCFAs), are revealed. A mouse model of hepatic fibrosis was built by the subcutaneous injection of CCl_4_. Meanwhile, Kae was administered by gavage at doses of 25, 50, and 100 mg/kg body weight. Serum biomarkers, liver histopathology, oxidative damage markers, and nuclear factor erythroid 2-related factor 2 (Nrf2)/kelch-like ECH-associated protein 1 (Keap1)/heme oxygenase 1 (HO-1) signaling were analyzed. AML12 hepatocytes were pretreated with Kae or SCFAs (acetate, propionate, butyrate) before H_2_O_2_-induced oxidative injury. The changes in gut microbiota and the levels of SCFAs were assessed via 16S rRNA sequencing and GC-MS, respectively. Kae effectively alleviated the destruction of the liver morphology and tissue structure, reduced the infiltration of inflammatory cells, collagen deposition in the liver, and the expression of fibrotic factors, and downregulated the oxidative stress level in the liver of mice with liver fibrosis by activating the Nrf2/Keap1/HO-1 pathway (*p* < 0.05 or 0.01). In vitro, Kae significantly mitigated H_2_O_2_-induced cytotoxicity and oxidative damage (*p* < 0.05 or 0.01). Furthermore, Kae restored gut microbiota diversity, increased beneficial genera (e.g., *Lactobacillus*), and elevated both intestinal and hepatic SCFA levels (*p* < 0.01). The discrepant SCFA pretreatment similarly protected AML12 cells by activating Nrf2 signaling (*p* < 0.05 or 0.01). Our research suggests that Kae could inhibit CCl_4_-induced liver fibrosis by restoring the levels of intestinal metabolite SCFAs to reduce oxidative damage.

## 1. Introduction

Liver fibrosis represents an abnormal wound repair response induced by multiple chronic liver injuries, ultimately evolving into liver cirrhosis and hepatocellular carcinoma [1]. Globally, the number of deaths due to liver cirrhosis and cancer is on the rise, accompanied by a high mortality rate [2]. Despite the advancements in understanding its pathogenesis, therapeutic options for reversing or halting fibrosis remain limited, with liver transplantation being the sole curative intervention for end-stage liver fibrosis, thereby highlighting the significance of seeking effective anti-hepatic fibrosis drugs and active ingredients [3].

A considerable number of studies have indicated that intestinal flora dysbiosis plays a crucial role in the progression of liver fibrosis [4]. The alterations in intestinal flora could modify the body’s oxidative stress levels [5]. It is also proven that the changes in flora could result in a deficiency of glycine within the intestine, reducing glutathione synthesis and thereby triggering oxidative stress responses in the body [6]. Compared with other cells, hepatocytes possess higher levels of antioxidant enzymes such as superoxide dismutase (SOD) and catalase (CAT). Under normal circumstances, superoxide is transformed into hydrogen peroxide by SOD, which is subsequently converted into non-toxic water by CAT [7]. However, when there is an excessive generation of reactive oxygen species (ROS) within the tissue or an inadequate antioxidant mechanism, it is prone to inducing an oxidative stress state, leading to lipid peroxidation of cell membranes, abnormal organelle functions, inflammatory reactions, oxidative damage to the nucleus, and triggering various signal cascades, resulting in abnormal liver function [7]. Additionally, metabolites produced by the intestinal flora, such as short-chain fatty acids (SCFAs) (predominantly butyrate, acetate, and propionate), exert protective effects on the liver, and some SCFAs also exhibit antioxidant properties [8,9].

**Figure 1:**
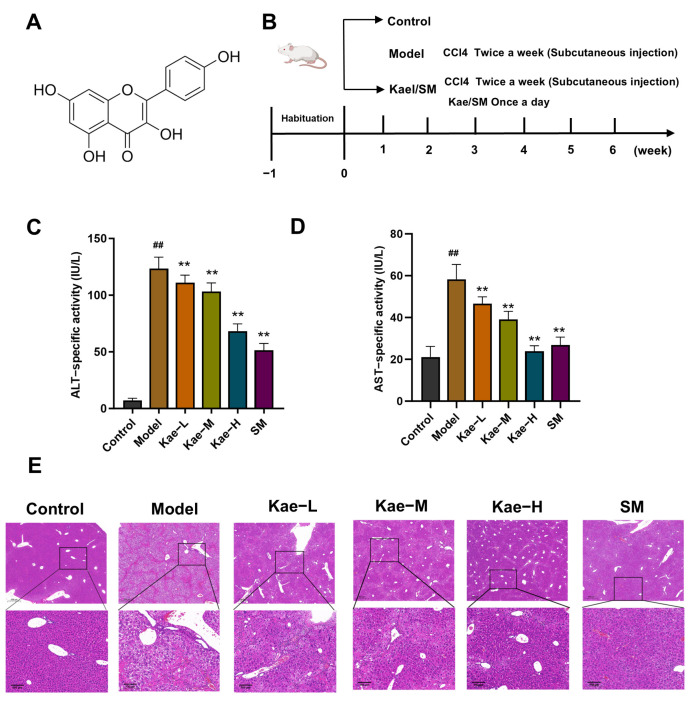
Kae inhibits CCl_4_-induced liver injury in mice. (**A**) The chemical structure of Kae. (**B**) Animal experimental research design. Serum concentrations of (**C**) ALT and (**D**) AST of mice in each group (mean ± SD, *n* = 8). (**E**) Representative hematoxylin and eosin (H&E). Sections of the left hepatic lobe in mice (scale bar 500 μm). All data are shown as the mean ± SD. ^##^, *p* < 0.01 vs. the control group. **, *p* < 0.01 vs. the model group. ALT, alanine aminotransferase; AST, aspartate aminotransferase; H&E, hematoxylin and eosin; Kae, kaempferol; Kae-L, low dose of Kae (25 mg/kg body weight); Kae-M, medium dose of Kae (50 mg/kg body weight); Kae-H, high dose of Kae (100 mg/kg body weight); SM, silymarin (300 mg/kg body weight); SD, standard deviation.

Kaempferol (Kae) is one of the most common flavonoids, derived from various traditional Chinese medicinal herbs and natural plants [10]. Kae has attracted the attention of researchers for its extensive pharmacological activities [11]. The molecular structure of Kae is shown in Figure 1A. Kae has been reported to protect against liver injury induced by carbon tetrachloride (CCl_4_), acetaminophen, and a high-fat diet [12,13,14]. Although previous studies have documented the hepatoprotective activity of Kae [15], Kae has poor absorption, and a large part of it is metabolized before entering the liver [16]. Researchers have revealed that gut microbiota is one of the targets for Kae to exert its biological activities [17]. Moreover, Kae has been proven to treat hepatic fibrosis in mice by modulating intestinal flora [18]. However, the association between the regulation effect of the intestinal microbiota of Kae and its anti-liver fibrosis effect has not yet been revealed.

The current study aims to reveal the mechanisms that allow Kae to inhibit hepatic fibrosis by remodeling the composition of intestinal flora, followed by increasing the content of beneficial SCFAs in the intestine tract, thereby reducing oxidative damage to liver cells. In this research, CCl_4_ was conducted to induce fibrotic reactions in the liver. The index of liver fibrosis, the composition of intestinal flora and SCFAs in the gut tract and the liver tissues, and the oxidative damage of hepatocytes were determined. Our research elucidates that Kae not only mitigates liver fibrosis by attenuating oxidative damage to hepatic cells but also achieves this effect through the regulation of SCFAs originating from the intestine that subsequently enter hepatic tissues.

## 2. Results

### 2.1. Kae Inhibits CCl_4_-Induced Mice Hepatic Injury and Fibrosis

Obvious liver injury was observed in mice exposed to carbon tetrachloride (CCl_4_), as evidenced by a significant increase in both alanine aminotransferase (ALT) and aspartate aminotransferase (AST) when comparing the model mice to the control group (Figure 1C,D, *p* < 0.01). Following the administration of various concentrations of Kae (25, 50, 100 mg/kg body weight) and the positive control medicine silymarin (SM) (300 mg/kg body weight), the levels of ALT and AST in the serum were significantly decreased (Figure 1C,D, *p* < 0.01), suggesting that Kae exerts an inhibitory effect on liver injury induced by CCl_4_. As revealed by hematoxylin and eosin (H&E) staining, the liver lobule structure of the mice in the control group was intact, and the hepatocytes were arranged orderly. In the model group, a considerable amount of inflammatory cell infiltration, hepatocyte damage, and necrosis in the liver tissues was observed. Both the intervention of Kae and SM could reduce inflammatory cell infiltration, restore the structure of the liver tissue and hepatocytes, and ameliorate the pathological damage of liver tissue (Figure 1E).

**Figure 2:**
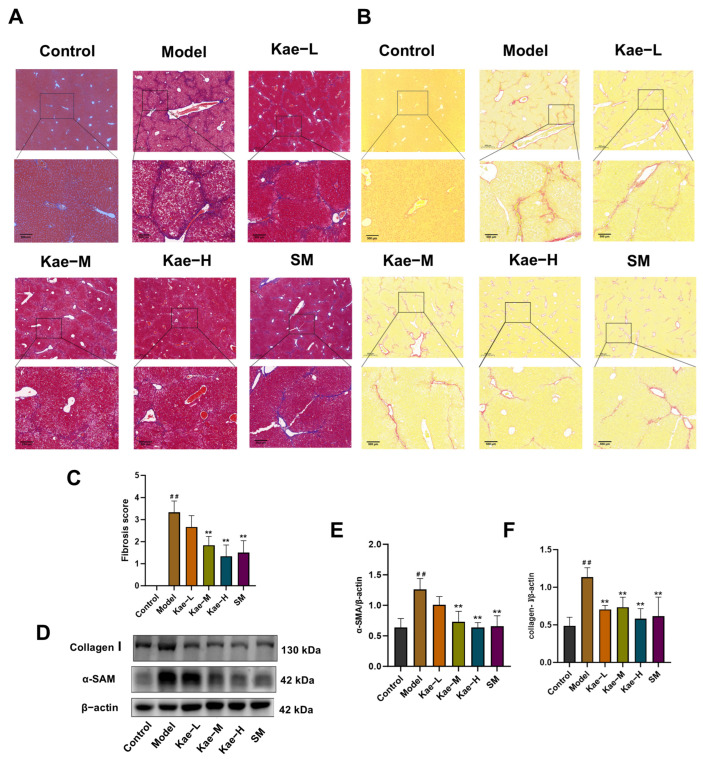
Kae inhibits CCl_4_-induced liver fibrosis in mice. The representative images selected from Masson’s (**A**) trichrome and Sirius red staining (**B**) (scale bar 500 μm). (**C**) The analysis of the fibrosis grade in Masson’s trichrome staining, *n* = 9. (**D**) The protein expression of α-SMA and Collagen I was treated with Kae and silymarin in mice. β-actin was used as the loading control. (**E**,**F**) Quantitative image analysis of (**D**) (*n* = 5). All data are shown as the mean ± SD. ^##^, *p* < 0.01 vs. the control group. **, *p* < 0.01 vs. the model group. α-SMA, alpha smooth muscle actin; Kae, kaempferol; Kae-L, low dose of Kae (25 mg/kg body weight); Kae-M, medium dose of Kae (50 mg/kg body weight); Kae-H, high dose of Kae (100 mg/kg body weight); SM, silymarin (300 mg/kg body weight); SD, standard deviation.

Histopathological analysis utilizing Masson’s and Sirius red staining indicated that the treatment with Kae and SM significantly mitigated CCl_4_-induced hepatic fibrotic changes, as evidenced by a reduction in fibrous septa formation and collagen deposition in comparison to the model group (Figure 2A–C). Moreover, Western blot analysis revealed that alpha smooth muscle actin (α-SMA) and Collagen I, the typical fibrotic markers, were significantly upregulated in the model group compared to the control group (Figure 2C–E, *p* < 0.01). Notably, Kae and SM treatment at varying concentrations dose-dependently attenuated these alterations. These findings demonstrate that Kae effectively inhibits CCl_4_-induced liver fibrosis by suppressing the profibrotic protein expression. The inhibitory effect of a high dose of Kae on liver fibrosis was comparable to that of SM.

### 2.2. Kae Attenuates Oxidative Stress in Liver Tissues of CCl_4_-Treated Mice

**Figure 3:**
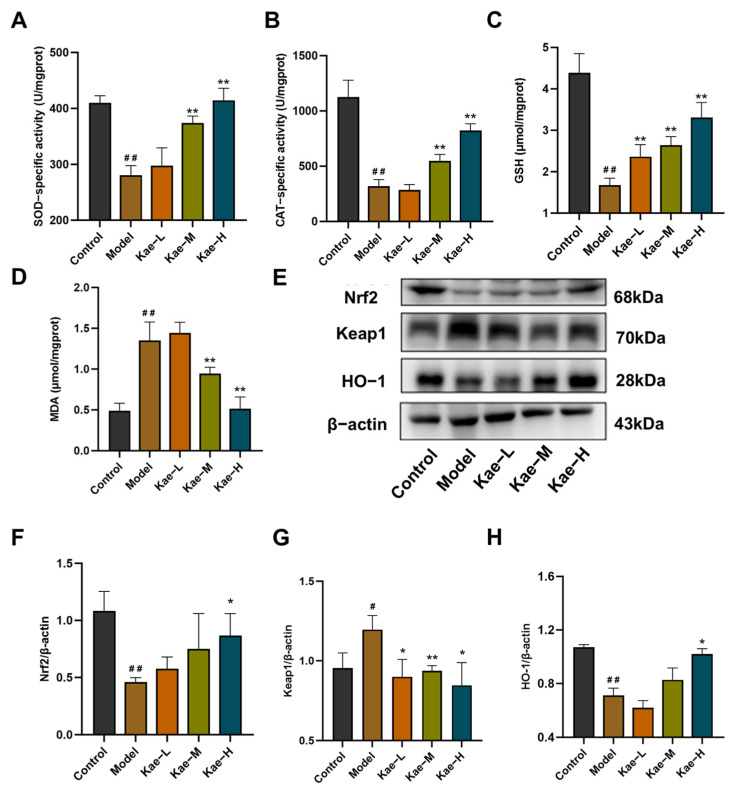
Kae attenuates oxidative stress in mice with CCl_4_-induced liver fibrosis. Comparative analysis of hepatic oxidative stress markers, (**A**) SOD, (**B**) CAT, (**C**) GSH, and (**D**) MDA (*n* = 6). (**E**) Representative Western blot of Nrf2, Keap1, and HO-1 protein. (**F**–**H**) Quantitative image analysis of (**E**) (*n* = 3). All data are shown as the mean ± SD. ^#^, *p* < 0.05, ^##^, *p* < 0.01 vs. the control group. *, *p* < 0.05, **, *p* < 0.01 vs. the model group. CAT, catalase; GSH, glutathione; MDA, malondialdehyde; SOD, superoxide dismutase; HO-1, heme oxygenase 1; Kae, kaempferol; Kae-L, low dose of Kae (25 mg/kg body weight); Kae-M, medium dose of Kae (50 mg/kg body weight); Kae-H, high dose of Kae (100 mg/kg body weight); Keap1, kelch-like ECH-associated protein 1; Nrf2, nuclear factor erythroid 2-related factor 2; SD, standard deviation.

Compared to those in the control group, the liver tissues of the fibrotic mice in the model group exhibited a significant decrease in the activities of antioxidant enzymes, including SOD (Figure 3A, *p* < 0.01) and CAT (Figure 3B, *p* < 0.01), as well as a reduction in the content of glutathione (GSH) (Figure 3C, *p* < 0.01) levels, accompanied by an observable increase in the lipid peroxidation marker malondialdehyde (MDA) (Figure 3D, *p* < 0.01). Notably, Kae treatment dose-dependently reversed these perturbations (*p* < 0.01).

Mechanistically, Western blot analysis revealed that Kae upregulated the Nrf2 antioxidant pathway. The expression of Nrf2 and its downstream target HO-1 in hepatic tissues were significantly upregulated in Kae-treated groups compared to the model group (Figure 3E,F,H, *p* < 0.05 or 0.01). As expected, the expression of Keap1 (Figure 3E,G, *p* < 0.05 or 0.01), a negative regulator of Nrf2, was also downregulated. These coordinated changes suggest that Kae alleviates oxidative damage by enhancing Nrf2-mediated antioxidant defenses.

### 2.3. Kae Alleviates Hydrogen Peroxide-Induced Oxidative Damage in AML12 Cells

**Figure 4:**
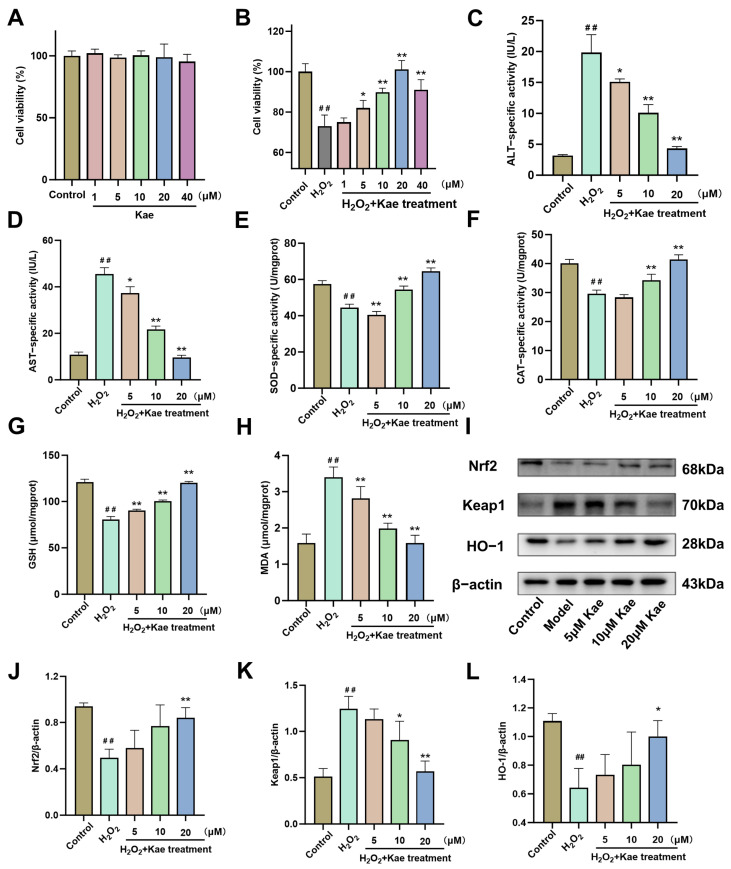
Kae alleviates H_2_O_2_-induced oxidative damage in AML12 cells. (**A**,**B**) CCK8 was used to determine the viability of AML12 cells treated with Kae and H_2_O_2_ (*n* = 6). (**C**,**D**) Concentrations of ALT (**C**) and AST (**D**) levels were treated with H_2_O_2_ (200 μM) and Kae (5, 10, 20 µM) (*n* = 3). Concentrations of SOD (**E**), CAT (**F**), GSH (**G**), and MDA (**H**). (**I**) Representative Western blot of Nrf2, Keap1, and HO-1 (*n* = 3). (**J**–**L**) Quantitative image analysis of (**I**) (*n* = 3). All data are shown as the mean ± SD. ^##^, *p* < 0.01 vs. the control group. *, *p* < 0.05, **, *p* < 0.01 vs. the H_2_O_2_ treatment group. ALT, alanine aminotransferase; AST, aspartate aminotransferase; CAT, catalase; GSH, glutathione; MDA, malondialdehyde; SOD, superoxide dismutase; HO-1, heme oxygenase 1; Kae, kaempferol; Kae-H, high dose of Kae (100 mg/kg body weight); Keap1, kelch-like ECH-associated protein 1; Nrf2, nuclear factor erythroid 2-related factor 2; SD, standard deviation.

The results of the CCK-8 assay indicated that Kae within the concentration range of 1 to 40 μM has no significant effect on the viability of hepatocytes in vitro (Figure 4A). Following that, H_2_O_2_ was conducted to establish an in vitro model of hepatocyte oxidative injury. Then, in H_2_O_2_-induced hepatocytes with oxidative damage, the pretreatment with Kae at the concentrations of 5–40 μM observed a dose-dependent rescue effect on cell viability (Figure 4B, *p* < 0.05 or 0.01). Moreover, the subsequent experiments were conducted using Kae at the concentrations of 5, 10, and 20 μM to assess the ALT and AST levels in hepatocytes. The results demonstrated that the pretreatment with Kae at these three concentrations significantly attenuated the H_2_O_2_-induced elevation of ALT and AST levels (Figure 4C,D, *p* < 0.05 or 0.01).

The subsequent studies demonstrated that Kae effectively restored the activities of SOD and CAT enzymes (Figure 4E,F, *p* < 0.01) and the contents of GSH (Figure 4G, *p* < 0.01), which had been diminished due to oxidative stress induced by H_2_O_2_. Additionally, Kae was observed to significantly reduce the levels of MDA (Figure 4H, *p* < 0.01). Western blot analysis revealed that, in comparison to the control cells, the expression of the Nrf2 protein was diminished in the H_2_O_2_-treated cells. However, the intervention with 20 μM Kae resulted in an increase in Nrf2 expression (Figure 4I,J, *p* < 0.01). Additionally, the expression of Keap1 was elevated in the H_2_O_2_ stimulated cells (*p* < 0.01) but was reduced in the cells treated with 10 and 20 μM Kae (Figure 4I,K, *p* < 0.01 or 0.05). Consistent with the changes in Nrf2 expression, the expression of HO-1 also increased in the cells treated with 20 μM Kae (Figure 4I,L).

### 2.4. Kae Restores Gut Microbiota Disorder in Liver Fibrosis Mice

**Figure 5:**
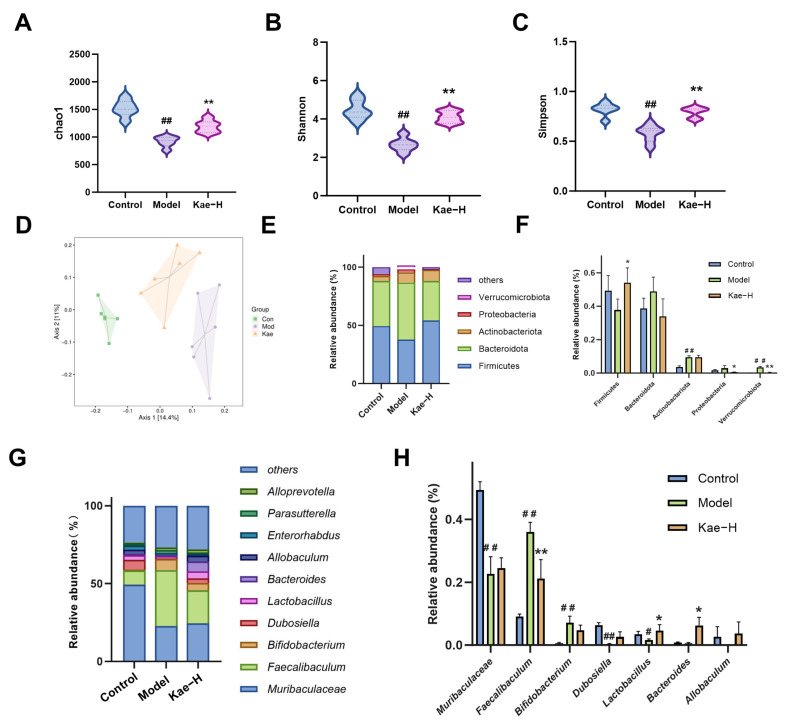
Kae restores gut microbiota disorder in liver fibrosis mice. (**A**–**C**) The Chao1, Shannon, and Simpson indices of the murine gut microbiota (*n* = 6). (**D**) Principal coordinate analysis (PCA) method for the structure of mouse gut microbiota. (**E**) Distribution of mouse gut microbiota at the phylum taxonomic level. (**F**) Comparison of differences between groups of (**E**). (**G**) Distribution of mouse gut microbiota at the taxonomic level of the genus. (**H**) Comparison of differences between groups of (**G**) (*n* = 6). All data are shown as the mean ± SD. ^#^, *p* < 0.05, ^##^, *p* < 0.01 vs. the control group. *, *p* < 0.05, **, *p* < 0.01 vs. the model group. Kae, kaempferol; Kae-H: high dose of Kae (100 mg/kg); SD, standard deviation.

The distribution of the gut microbiota of mice in the control group, the model group, and the Kae-H group was analyzed by 16S rRNA sequencing. The richness, diversity, and evenness of the intestinal microbiota were analyzed by the Chao1, Shannon, and Simpson indexes, respectively. Compared to the control group, the intestinal flora of the mice in the model group exhibited a significant reduction in the Chao1, Shannon, and Simpson indexes. However, subsequent treatment with Kae restored all these indices (Figure 5A–C, *p* < 0.01). The principal component analysis showed that the samples from the model group were distantly located from those of the control group, while the samples from the Kae-treated group were positioned between the control and model groups (Figure 5D). This result suggests that after Kae administration, the gut microbiota structure in the mice gradually shifted towards that of the control group, indicating a partial restoration of the microbial community. The analysis results of the structure of the gut microbiota showed that the intestinal microbiota of mice in each detected group mainly consisted of Firmicutes, Bacteroidota, Actinobacteriota, Proteobacteria, and Verrucomicrobia, accounting for more than 90% of the intestinal microbiota at the phylum level (Figure 5E). Compared to the control group, the relative abundance of Firmicutes was significantly reduced in the model group, while the relative abundances of the other four phyla increased. In contrast to the model group, the relative abundance of Firmicutes was increased in the Kae-H group, while the relative abundances of Bacteroidota and Proteobacteria were decreased (Figure 5F, *p* < 0.01). At the genus level, the composition of the gut microbiota of mice in each detected group mainly included *Muribaculaceae*, *Faecalibaculum*, *Bifidobacterium*, *Dubosiella*, *Lactobacillus*, *Bacteroides*, *Allobaculum*, etc. (Figure 5G). Compared with the control group, the relative abundances of *Faecalibaculum* and *Bifidobacterium* were increased in the model group, while the relative abundances of *Muribaculaceae*, *Dubosiella*, *Lactobacillus*, *Bacteroides*, and *Allobaculum* was decreased. After Kae intervention, the relative abundances of *Faecalibaculum* and *Bifidobacterium* were downregulated, while the relative abundances of *Dubosiella*, *Lactobacillus*, *Bacteroides*, and *Allobaculum* were upregulated (Figure 5H, *p* < 0.01). The above analysis revealed that Kae could regulate the disturbance of intestinal flora while playing an anti-fibrosis role.

### 2.5. Kae Regulates SCFAs in Both Intestinal Contents and Liver Tissues

**Figure 6:**
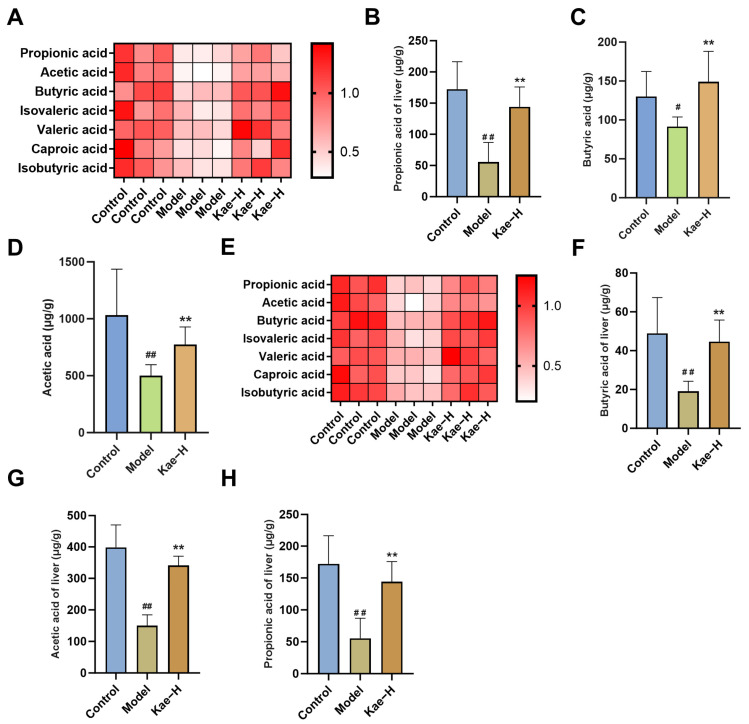
Kae regulates SCFAs in intestinal contents and liver tissues. (**A**) Heat map of short-chain fatty acids in intestinal soluble matter. Comparison of differential SCFAs in the intestines of mice in each group: propionic acid (**B**), butyric acid (**C**), and acetic acid (**D**) (*n* = 6). (**E**) Heat map of short-chain fatty acids in liver tissue. Comparison of differential SCFAs in the liver of mice in each group: propionic acid (**F**), butyric acid (**G**), and acetic acid (**H**) (*n* = 6). All data are shown as the mean ± SD. ^#^, *p* < 0.05; ^##^, *p* < 0.01 vs. the control group. **, *p* < 0.01 vs. the model group. Kae, kaempferol; Kae-H, high dose of Kae (100 mg/kg body weight); SCFA, short-chain fatty acid; SD, standard deviation.

According to the detection of gas chromatography-mass spectrometry (GC-MS), there were obvious differences among the composition of SCFAs in different detected groups. Compared with the control group, the contents of acetic acid, propionic acid, and butyric acid in the intestines of mice in the model group decreased. The results demonstrated that after Kae intervention, the decreased levels of acetate, propionate, and butyrate were restored (Figure 6A–D, *p* < 0.01). Moreover, the levels of SCFAs in liver tissues were quantified, and their variations mirrored the trends observed in the intestinal SCFA profiles of the corresponding cohorts (Figure 6E–H, *p* < 0.01). These results indicate a potential mechanism that Kae regulates intestinal SCFAs, which enter liver tissue through enterohepatic circulation and thus interfere with liver fibrosis.

### 2.6. SCFAs Attenuate H_2_O_2_-Induced Oxidative Damage in AML12 Cells

**Figure 7:**
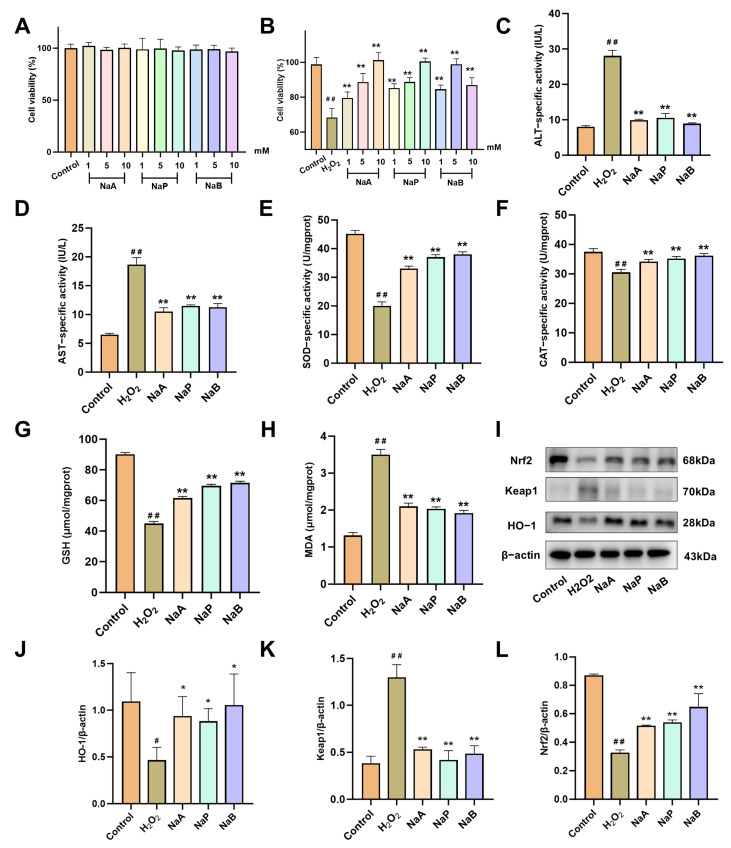
SCFAs attenuate H_2_O_2_-induced oxidative damage in AML12 cells. (**A**,**B**) CCK8 was used to determine the viability of AML12 cells treated with NA, NP, NB, and H_2_O_2_ at indicated concentrations (*n* = 6). (**C**,**D**) Concentrations of ALT (**C**) and AST (**D**) levels were treated with H_2_O_2_ (200 μM) and NA (10 mM), NaP (10 mM), and NaB (5 mM) (*n* = 3). Concentrations of SOD (**E**), CAT (**F**), GSH (**G**), and MDA (**H**) (*n* = 6). (**I**) Representative Western blot of Nrf2, Keap1, and HO-1. (**J**–**L**) Quantitative image analysis of (**I**) (*n* = 6). All data are shown as the mean ± SD. ^#^, *p* < 0.05; ^##^, *p* < 0.01 vs. the control group. *, *p* < 0.05; **, *p* < 0.01 vs. the H_2_O_2_ treatment group. ALT, alanine aminotransferase; AST, aspartate aminotransferase; CAT, catalase; GSH, glutathione; MDA, malondialdehyde; SOD, superoxide dismutase; HO-1, heme oxygenase 1; Kae, kaempferol; Keap1, kelch-like ECH-associated protein 1; NaA, sodium acetate; NaB, sodium butyrate; NaP, sodium propionate; Nrf2, nuclear factor erythroid 2-related factor 2; SD, standard deviation.

Based on the above findings, the differential SCFAs induced by Kae intervention, sodium acetate (NaA), sodium propionate (NaP), and sodium butyrate (NaB), were pretreated to the hepatocyte injury model induced by H_2_O_2_. The CCK8 assay results indicated that these three SCFA salts did not exhibit any significant impact on the viability of the AML12 cells (Figure 7A). In contrast, in H_2_O_2_-induced oxidative stress-damaged AML12, the pretreatment with NaA, NaP, and NaB demonstrated a significant protective effect, partially rescuing cell viability (Figure 7B, *p* < 0.01). The above three SCFA salts that exhibited the most effective protective effects (10 mM NaA, 10 mM NaP, and 5 mM NaB) markedly downregulated the levels of ALT and AST in H_2_O_2_-intervened AML12 cells (Figure 7C,D, *p* < 0.01). In alignment with previous research, these SCFAs were found to restore the activities of SOD and CAT and upregulate the level of GSH while downregulating the content of MDA (Figure 7E–H, *p* < 0.01). Western blot analyses further revealed that the detected SCFAs restored the expression of Nrf2 and HO-1 proteins while concurrently reducing the expression of Keap1 (Figure 7I–L, *p* < 0.05 or 0.01). These results support that the SCFAs affected by Kae may activate the Nrf2/Keap1 signaling pathway, thereby enhancing the cellular antioxidant response and safeguarding hepatocytes against oxidative damage.

## 3. Discussion

Liver fibrosis is distinguished by the excessive deposition of the extracellular matrix and progressive liver dysfunction, arising from various insults including toxic damage, alcohol abuse, viral infections, and metabolic disorders. Despite the advances in elucidating its molecular mechanisms, current therapeutic strategies remain limited, with a pressing need for agents capable of targeting both fibrotic pathways and associated hepatocyte damage.

In recent years, research has indicated that the disorder of intestinal flora and its metabolites plays a pivotal role in the progression of liver fibrosis [19]. Modern research has confirmed that the liver and gut are not only anatomically interconnected but also functionally closely related [20]. The gastrointestinal tract and liver engage in the exchange of metabolites, such as SCFAs and bile acids, via the enterohepatic axis [21]. A recent study has demonstrated that those exchanged metabolites could mitigate tissue injury [22].

Although the mechanisms leading to liver fibrosis are not fully elucidated, it is well established that hepatocyte damage induced by oxidative stress is a critical process driving liver injury and initiating fibrosis [23]. This condition corresponds to an imbalance between intracellular pro-oxidant and antioxidant factors, resulting in the excessive production of oxidation products [4]. After the liver is stimulated by damaging factors, hepatocytes could stimulate hepatic stellate cells through multiple pathways, thereby initiating liver fibrosis [24]. Hepatocytes are often used as the target cells of drugs against liver fibrosis for research, especially in the mechanism of oxidative stress [25]. As a commercial cell line of mouse hepatocytes, the AML12 cell line was widely used in the above-mentioned research [26].

Nrf2 is a pivotal transcriptional regulator of cellular antioxidant responses, fundamentally contributing to the maintenance of redox homeostasis and safeguarding cells against oxidative damage. The Nrf2/HO-1 pathway has been extensively implicated in mitigating oxidative stress-associated pathologies, including liver fibrosis, by enhancing cellular defense mechanisms and reducing the accumulation of oxidants [27]. Recent evidence has underscored the significance of the gut–liver axis as a key mediator in fibrosis progression, where gut microbiota dysbiosis and altered SCFA composition exacerbate hepatic oxidative stress and inflammation.

Kae, a flavonoid compound widely present in plants, exhibits significant anti-inflammatory, antioxidant, and hepatoprotective pharmacological activities [28]. Its hepatoprotective effects have been validated in various models [16]. This research has demonstrated that Kae significantly reduces serum ALT and AST levels in CCl_4_-induced liver injury mice, alleviates inflammatory cell infiltration, restores liver tissue and the hepatocyte structure, and improves liver histopathological damage. Furthermore, Kae effectively inhibits the progression of CCl_4_-induced hepatic fibrosis as evidenced by the downregulated expression of fibrosis marker proteins, α-SMA and Collagen I. Similar to SM, the hepatoprotective effects of Kae primarily rely on its potent antioxidant activity [29]. These findings suggest that Kae holds potential therapeutic value for the treatment of liver injury and subsequent fibrosis. However, the mechanisms of Kae against liver fibrosis still need to be further revealed.

Previous research has indicated that Kae may mitigate liver injury in mice by activating the Nrf2 signaling pathway and diminishing oxidative damage to liver cells [29]. Our studies also showed consistent results. However, given the well-documented poor water solubility and bioavailability of Kae [16], it is suggested that Kae may not directly affect the liver tissues. Several studies have indicated that intestinal flora is a potential target for Kae to exert its biological functions [17,18]. In the hepatic fibrosis mouse model we developed, the Kae intervention demonstrated a significant improvement in the disturbances of the intestinal flora associated with fibrosis. Moreover, there is a notable reduction in the Firmicutes/Bacteroidetes ratio, which is believed to be closely associated with the amelioration of liver fibrosis [30]. At the genus level, the increased abundance of the genus *Dubosiella* and *Lactobacillus* has also been demonstrated to correlate with improvements in liver damage arising from various etiologies [31]. Notably, the changes in *Clostridium butyricum* were consistent with subsequent alterations in SCFAs across the three groups analyzed.

SCFAs, a group of organic fatty acids containing 1 to 6 carbon atoms, serve as crucial mediators through which the intestinal microbiota engages in physiological functions. Among these, acetate, propionate, and butyrate are the most common, accounting for 90–95% of the total SCFAs produced in the gastrointestinal tract [32]. It has been reported that the supplementation of SCFAs, which are markedly reduced in the intestines of individuals with liver fibrosis, may significantly ameliorate the progression of liver disease [33,34]. In investigating the mechanism of SCFA intervention in liver disease, researchers have predominantly concentrated on its protective effects on the intestinal barrier [35]. SCFAs serve as the primary energy source for colonocytes, and previous studies have demonstrated that SCFAs help maintain the intestinal barrier and reduce intestinal permeability by reducing local colonic inflammation and producing antimicrobial peptides [8]. The interventions aimed at regulating SCFAs have the potential to mitigate or arrest the progression of liver disease. This is achieved by attenuating immune-mediated damage resulting from the portal dissemination of pathogen-associated molecular patterns and bacterial endotoxins [9,36]. In recent years, there has been a growing research emphasis on the SCFAs translocated to the liver tissues [37,38].

To elucidate the regulatory mechanism of Kae on hepatic fibrosis and whether it relates to the modulation of SCFAs, SCFAs in murine intestinal contents and hepatic tissues were quantitatively analyzed by GC-MS. In this research, the intervention of Kae was demonstrated to elevate the concentrations of acetic acid, propionic acid, and butyric acid in both the intestinal tracts and liver tissues of mice afflicted with liver fibrosis. Further study results revealed that these SCFAs could significantly reduce the damage caused by the H_2_O_2_ stimulation of liver cells. The above evidence amply indicates that the regulation of intestinal flora and its SCFAs is part of the mechanisms by which Kae protects hepatocytes from oxidative stress and thus prevents liver fibrosis. Future research is planned to investigate the regulatory role of Kae in the transport of SCFAs to the liver. Concurrently, the identification of target sites for NaB in the protection of hepatic cells warrants further exploration.

The above results position Kae as a translational candidate for bridging gut–liver axis modulation for treating liver fibrosis. Current antifibrotic drugs, such as pirfenidone, have limitations due to side effects and variable efficacy. Kae has a favorable safety profile, supported by its presence in dietary sources, and may enable long-term prophylactic use in high-risk populations. Moreover, the dose-dependent effects observed provide a rationale for human equivalent dosing in future trials. We propose that Kae could be trialed as a complementary therapy alongside lifestyle interventions to halt fibrosis progression.

## 4. Materials and Methods

### 4.1. Reagents

Kae (>98%) was purchased from Pusi Biotechnology Co., Ltd. (PS011599, Chengdu, Sichuan, China). Silymarin (SM, >98%) was obtained from Maclean Biochemical Technology (S304299, Shanghai, China). CCl_4_ was purchased from Sinopharm Chemical Reagent Co., Ltd. (20241010, Shanghai, China). Peanut oil was purchased from Luhua Co., Ltd. (05638, Yantai, Shandong, China). Acetic acid, propionic acid, butyric acid, isobutyric acid, valeric acid, isovaleric acid, and 4-methylvaleric acid were obtained from Sigma (A6283-100ML, 402907-100ML, B103500-100ML, 58360-100ML, 240370-100ML, 129542-100ML, and 277827-5G, St. Louis, MI, USA). Sodium acetate (NaA), sodium propionate (NaP), and sodium butyrate (NaB) were purchased from Shanghai Co., Ltd. (S118648-100g, S100122-100g, and S102956-4g, Aladdin Bio-Chem Technology, Shanghai, China). The kits of CCK-8, ALT, AST, SOD, GSH, MDA, and CAT were purchased from Elabscience (E-CK-A362, E-BC-K235-M, E-BC-K236-M, E-BC-K019-M, E-BC-K030-M, E-BC-K031-M, and E-BC-K025-M, Wuhan, Hubei, China). RIPA, BCA kit, and NcmBlot blocking buffer were purchased from NCM Biotech (WB3100, WB6501, and P30500, Suzhou, Jiangsu, China). ECL was purchased from Biosharp (BL520B, Hefei, Anhui, China). Anti-α-SMA, anti-Collagen I, anti-Nrf2, anti-Keap1, anti-HO-1, and anti-β-actin were purchased from Proteintech (67735-1-lg, 14695-1-AP, 16396-1-AP, 60027-1-lg, 10701-1-AP, and 66009-1-lg, Wuhan, Hubei, China). HRP Goat Anti-Mouse Ig (H+L) and HRP Goat Anti-Rabbit Ig (H+L) were purchased from Elabscience (E-AB-1001 and E-AB-1003, Wuhan, Hubei, China).

### 4.2. Animals

Male-specific pathogen-free (SPF) C57BL/6 mice, 6–8 weeks of age (20.0 ± 2.0 g), were purchased from Hunan Slike Jinda Animal Co., Ltd. (Changsha, Hunan, China), license No. SCXK (Xiang) 2021-0002. The animals were kept under SPF conditions in the Animal Experiment Center of Hunan University of Chinese Medicine, maintaining a temperature of 24 ± 2 °C, a relative humidity of 50–60%, and a 12 h cycle of day and night. All experimental mice were housed in standard polypropylene cages with four mice per cage and were provided to obtain standard food and purified water unrestrictedly. All animals received humane care, and the research protocol was approved by the Animal Ethics Committee of the Hunan University of Chinese Medicine (Ethical review NO.: HNUCN11-2409-212).

### 4.3. Experimental Design

After 7 days of adaptive feeding, 48 mice were randomly divided into six groups (*n* = 8 mice per group): the control group (Control), the model group (Model), the low-dose Kae treatment group (Kae-L), the medium-dose Kae treatment group (Kae-M), the high-dose Kae treatment group (Kae-H), and the positive control group.

As we described in the previous paper [30], mice in all groups, except the control group received subcutaneous injections of 20% CCl_4_ (*v*/*v*) in a peanut oil solution at a dose of 10 mL/kg body weight twice weekly for 6 weeks to establish the liver fibrosis model. Mice in the control group were administered an equivalent volume of peanut oil alone at the same time points. Referring to previous research on the pharmacological activity of Kae [39], and starting from the first day of modeling, the Kae-treated groups (Kae-L, Kae-M, and Kae-H) were intragastric administrated with Kae at 25, 50, and 100 mg/kg body weight once a day, respectively. Mice in the positive control group received SM (300 mg/kg body weight once a day) via oral gavage, with a dosing volume of 10 mL/kg body weight. Concurrently, mice in both the control and the model groups were administered an equal volume of the drug vehicle during the same period.

By the end of the sixth week, after the last intervention, all mice were subjected to fast for one day. Then, all the experimental mice were sacrificed after orbital blood extraction and subsequent anesthesia with pentobarbital sodium. The liver tissues and intestinal contents were collected for further analysis. The animal experiment design is shown in Figure 1B.

### 4.4. Histological Analysis

Part of the left lobe of mouse liver tissue was fixed in 10% neutral formaldehyde for 48 h, embedded in paraffin, and sectioned into slices with a thickness of 4 μm. The tissue sections after dewaxing were subjected to H&E, Masson, and Sirius red staining as follows for histological examination. H&E staining: The sections were stained by hematoxylin for a duration of 5 min, followed by rinsing under running tap water to achieve bluing. Subsequently, the sections were stained with eosin for two minutes. The samples were then dehydrated through a graded series of ethanol concentrations, cleared using xylene, and ultimately mounted with neutral balsam. Masson staining: The tissue sections underwent staining with Weigert’s iron hematoxylin for 10 min for nuclear visualization, followed by Ponceau acid fuchsin for 5 min for cytoplasmic staining. Then, the slices were subjected to differentiation using a phosphomolybdic acid solution for 5 min, after which the sections were stained with aniline blue for collagen detection for 5 min. Subsequently, the sections were rinsed in 1% acetic acid and finally dehydrated and mounted following the operation of H&E staining. Sirius red staining: The sections were stained with a 0.1% Sirius red solution for 1 h under light-protected conditions, briefly differentiated in 0.5% acetic acid, and then dehydrated and mounted following the operation of H&E staining. Three mice were taken from each group for sectioning, and three fields of view were taken from each mouse. Each microscopic field was examined under a light microscope. The degree of fibrosis was graded by the Ishak scoring system [40].

### 4.5. 16S rRNA Sequencing

The intestinal contents of the mice in the control group, the model group, and the Kae-H group were extracted for DNA. The variable regions of the rRNA gene or specific gene fragments were amplified by PCR [41]. The amplified products were purified and recovered using magnetic beads and then quantified by fluorescence. The TruSeq Nano DNA LT Library Prep Kit from Illumina was used to prepare the sequencing libraries, which were then sequenced by high-throughput sequencing.

### 4.6. Detection of SCFA Levels in Intestinal Contents and Liver Tissues

Appropriate amounts of the intestinal contents and hepatic tissues of the mice in the control group, the model group, and the Kae-H group were taken into 1.5 mL centrifuge tubes. Subsequently, 500 μL of water was added to homogenize for 1 min, followed by centrifugation at 4 °C for 10 min at 12,000 rpm. A volume of 200 μL of the supernatant was extracted with 100 μL of 15% phosphoric acid, 20 μL of a 375 μg/mL 4-methylvaleric acid solution as an internal standard, and 280 μL of ether. Subsequently, the samples underwent vortex mixing for 1 min and were centrifuged again at 4 °C for 10 min at 12,000 rpm. The resulting supernatant was then transferred into a vial for subsequent gas chromatography-mass spectrometry (GC-MS) analysis [42].

The GC analysis was performed using a Trace 1310 GC system (Thermo Fisher Scientific, Waltham, MA, USA). The system was configured with an Agilent HP-INNOWAX capillary column (30 m × 0.25 mm ID × 0.25 μm), utilizing helium as the carrier gas at a flow rate of 1 mL/min. A sample injection was performed in split mode with a ratio of 10:1, utilizing an injection volume of 1 μL and an injector temperature set at 250 °C. The ion source and mass spectrometer (MS) transfer line temperatures were maintained at 300 °C and 250 °C, respectively. The column temperature was programmed to increase from an initial temperature of 90 °C to 120 °C at a rate of 10 °C/min, followed by an increase to 150 °C at 5 °C/min, and finally to 250 °C at 25 °C/min, where it was held constant for 2 min. Metabolite analysis was performed using an ISQ 7000 mass spectrometer (Thermo Fisher Scientific, Waltham, MA, USA) operating in electron impact ionization mode. Single ion monitoring mode was employed with an electron energy of 70 eV.

### 4.7. Cell Culture

The AML12 cell line (mouse hepatocytes) and its special medium were obtained from Pricella (CM-0602, Pricella Wuhan, Hubei, China). The cells of passage 5–20 in the logarithmic growth phase were inoculated in cell culture plates at 37 °C with 5% CO_2_. The AML12 cell special medium contained 5, 10, and 20 μM Kae or 10 mM NaA, 10 mM NaP, and 5 mM NaB were added for 6 h of intervention. After that, the original medium in the wells was discarded again, and the basal medium and 200 μM H_2_O_2_ were added to induce oxidative damage.

### 4.8. CCK-8 Assay

The AML12 cells were seeded into 96-well plates until they reached proper confluence. Cell viability was analyzed by a CCK-8 kit (E-CK-A362, Wuhan, China).

### 4.9. Western Blot Analysis

The administrated hepatic tissues or cells were lysed in a RIPA lysis buffer containing protease and phosphatase inhibitors for 30 min and were then centrifuged at 13,300 rpm at 4 °C for 10 min. The supernatant was obtained, and the protein concentration was determined by a BCA kit. The proteins were separated by SDS-PAGE and transferred to polyvinylidene difluoride membranes (ISEQ00010, Millipore, St. Louis, MI, USA). After being blocked in an NcmBlot blocking buffer for 20 min at room temperature, the membranes were incubated with primary antibodies overnight at 4 °C. The membranes were incubated with HRP-conjugated secondary antibodies for 1 h at room temperature. After that, the immunoreactivity was detected by ECL, and the band intensities were quantified using ImageJ 1.8.0 software.

### 4.10. Biochemical Kits

ALT and AST levels of animal serum and AML12 cells after intervention were measured by commercial reagent kits, according to the manufacturer’s instructions. Then, the levels of oxidative stress biomarkers, including SOD, GSH, MDA, and CAT were measured by commercial reagent kits according to the manufacturer’s instructions.

### 4.11. Statistical Analysis

Statistical analyses were performed using GraphPad Prism 8.0 software. Prior to analysis, data normality was assessed. Normally distributed data are expressed as the mean ± standard deviation (SD). For comparisons of multiple independent groups, homogeneity of variance was tested, followed by one-way ANOVA. Post hoc tests (LSD for equal variances or Tamhane’s T2 for unequal variances) were selected based on the homogeneity of variance results. Non-normally distributed data were analyzed using non-parametric tests. A *p*-value < 0.05 was considered statistically significant.

## 5. Conclusions

Kae ameliorates CCl_4_-induced liver fibrosis by restoring the levels of gut microbiota-derived metabolites, particularly SCFAs, and subsequently alleviates oxidative stress. This mechanism highlights the potential of Kae as a therapeutic agent for liver fibrosis through the modulation of the gut–liver axis and the enhancement of antioxidant defenses.

## Data Availability

The data that support the findings of this study are available from the corresponding authors upon reasonable request.
